# *N*,*N*′-Bis[(*E*)-(2-chloro-8-methyl­quinolin-3-yl)methyl­idene]ethane-1,2-diamine

**DOI:** 10.1107/S160053681004540X

**Published:** 2010-11-10

**Authors:** R. Prasath, P. Bhavana, Seik Weng Ng, Edward R. T. Tiekink

**Affiliations:** a Chemistry Group, BITS, Pilani - K. K. Birla Goa Campus, Goa 403 726, India; b Department of Chemistry, University of Malaya, 50603 Kuala Lumpur, Malaysia

## Abstract

The complete mol­ecule of the title compound, C_24_H_20_Cl_2_N_4_, is generated by a crystallographic inversion centre. A kink in the mol­ecule is evident [C—N—C—C torsion angle = −147.0 (3)°] owing to the twist in the central ethyl­ene bridge. Further, there is a small twist between the imine [N=C = 1.267 (3) Å] and quinoline residues [N—C—C—C = −12.4 (4)°]. In the crystal, a combination of π–π [pyridine–benzene centroid–centroid distance = 3.5670 (14) Å] and C—H⋯N contacts leads to supra­molecular chains propagating in [010].

## Related literature

For background to the photophysical properties of Schiff base complexes derivativatized with quinoline residues, see: Liu *et al.* (2010 ▶).
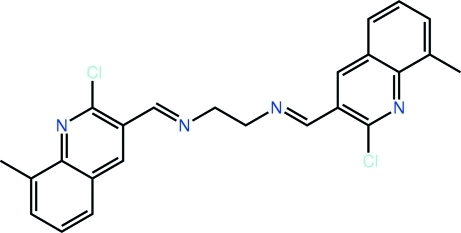


## Experimental

### 

#### Crystal data

C_24_H_20_Cl_2_N_4_
*M* *_r_* = 435.34
Monoclinic, 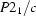

*a* = 18.363 (2) Å
*b* = 3.9494 (5) Å
*c* = 14.1726 (19) Å
β = 99.056 (2)°
*V* = 1015.0 (2) Å^3^
*Z* = 2
Mo *K*α radiation
μ = 0.34 mm^−1^
*T* = 100 K
0.30 × 0.15 × 0.05 mm

#### Data collection

Bruker SMART APEX diffractometer
Absorption correction: multi-scan (*SADABS*; Sheldrick, 1996 ▶) *T*_min_ = 0.756, *T*_max_ = 0.862
8859 measured reflections
2324 independent reflections
1863 reflections with *I* > 2σ(*I*)
*R*_int_ = 0.055

#### Refinement

*R*[*F* ^2^ > 2σ(*F* ^2^)] = 0.050
*wR*(*F* ^2^) = 0.140
*S* = 1.05
2324 reflections
137 parameters
H-atom parameters constrained
Δρ_max_ = 0.72 e Å^−3^
Δρ_min_ = −0.46 e Å^−3^

### 

Data collection: *APEX2* (Bruker, 2008 ▶); cell refinement: *SAINT* (Bruker, 2008 ▶); data reduction: *SAINT*; program(s) used to solve structure: *SHELXS97* (Sheldrick, 2008 ▶); program(s) used to refine structure: *SHELXL97* (Sheldrick, 2008 ▶); molecular graphics: *ORTEP-3* (Farrugia, 1997 ▶) and *DIAMOND* (Brandenburg, 2006 ▶); software used to prepare material for publication: *publCIF* (Westrip, 2010 ▶).

## Supplementary Material

Crystal structure: contains datablocks global, I. DOI: 10.1107/S160053681004540X/hb5727sup1.cif

Structure factors: contains datablocks I. DOI: 10.1107/S160053681004540X/hb5727Isup2.hkl

Additional supplementary materials:  crystallographic information; 3D view; checkCIF report

## Figures and Tables

**Table 1:** Hydrogen-bond geometry (Å, °)

*D*—H⋯*A*	*D*—H	H⋯*A*	*D*⋯*A*	*D*—H⋯*A*
C1—H1a⋯N1^i^	0.99	2.59	3.299 (3)	128

Symmetry code: (i) 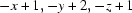.

**Fig. 1.**
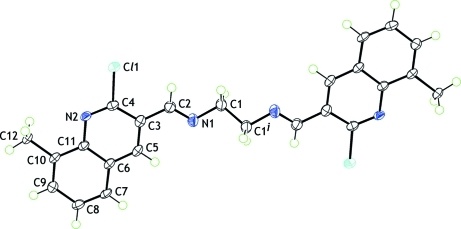
Molecular structure showing displacement ellipsoids at the 50% probability level. Symmetry operation i: 1 - x, 1 - y, 1 - z.

**Fig. 2.**
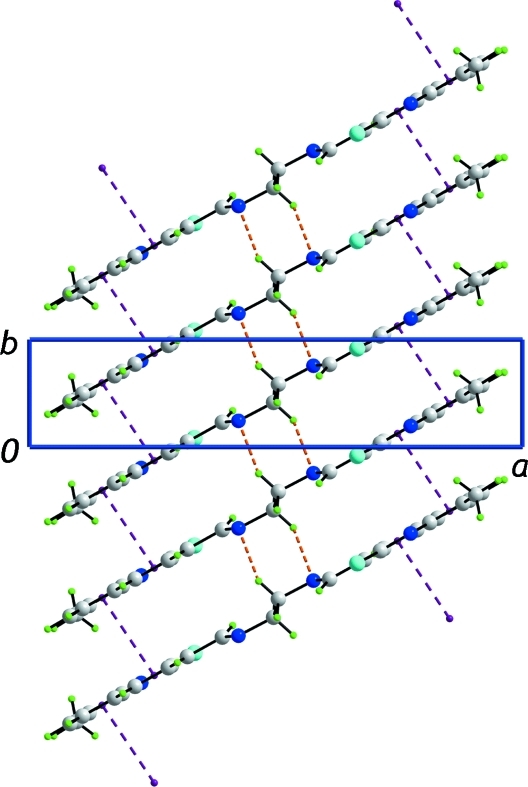
A view of the supramolecular chain along the b axis mediated by π–π and C—H···N contacts are shown as purple and orange dashed lines, respectively.

**Fig. 3.**
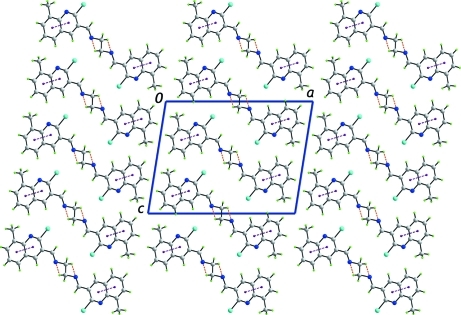
Stacking of chains in the crystal structure. The π–π and C—H···N contacts are shown as purple and orange dashed lines, respectively.

## supplementary crystallographic information

### Comment

Interest in the title compound arises as a result of the recent report of the photophysical properties of Schiff base complexes derivativatized with quinoline residues (Liu *et al.*, 2010). The title molecule, Fig. 1, is disposed about a centre of inversion so that the pendent quinoline groups are co-planar. Owing to the twist in the central ethylene bridge, there is a kink in the molecule as manifested in the value of the C2—N1—C1—C1^i^ torsion angle of -147.0 (3) °; *i*: 1 - *x*, 1 - *y*, 1 - *z*. There is a smaller twist between the imine [N1═C2 = 1.267 (3) Å] and quinoline residues as seen in the N1—C2—C3—C5 torsion angle of -12.4 (4) °.

The crystal packing is dominated by weak π–π and C—H···N contacts that lead to the formation of a supramolecular chain along the *b* axis, Fig. 2. The π–π contacts occur between translationally related quinoline rings [ring centroid(N2,C3–C6,C11)···ring centroid(C6–C11)^ii^ = 3.5670 (14) Å with an angle of inclination = 0.41 (11) ° for *ii*: *x*, -1 + *y*, *z*] and the C—H···N contacts occur between the methylene-H and imine-N1 atoms, Table 1. Chains pack as shown in Fig. 3.

### Experimental

A mixture of 2-chloro-3-formyl-8-methylquinoline (0.2 g, 1 m*M*) and ethylenediamine (0.03 ml, 0.5 m*M*) was stirred in dichloromethane for 3 h at room temperature. The solvent from the reaction mixture was removed under reduced pressure. The resulting solid was dried and purified by column chromatography using a 1:1 mixture of ethyl acetate and hexane. Recrystallization was by slow evaporation of a dichloromethane solution which yielded yellow prisms of (I). Yield: 65%. *M*.pt. 475–477 K.

### Refinement

Carbon-bound H-atoms were placed in calculated positions (C—H 0.95 to 0.99 Å) and were included in the refinement in the riding model approximation, with *U*_iso_(H) set to 1.2–1.5*U*_equiv_(C).

### Crystal data

**Table:**

C_24_H_20_Cl_2_N_4_	*F*(000) = 452
*M**_r_* = 435.34	*D*_x_ = 1.424 Mg m^−^^3^
Monoclinic, *P*2_1_/*c*	Mo *K*α radiation, λ = 0.71073 Å
Hall symbol: -P 2ybc	Cell parameters from 1737 reflections
*a* = 18.363 (2) Å	θ = 2.9–28.1°
*b* = 3.9494 (5) Å	µ = 0.34 mm^−^^1^
*c* = 14.1726 (19) Å	*T* = 100 K
β = 99.056 (2)°	Prism, yellow
*V* = 1015.0 (2) Å^3^	0.30 × 0.15 × 0.05 mm
*Z* = 2	

### Data collection

**Table:**

Bruker SMART APEX diffractometer	2324 independent reflections
Radiation source: fine-focus sealed tube	1863 reflections with *I* > 2σ(*I*)
graphite	*R*_int_ = 0.055
ω scans	θ_max_ = 27.5°, θ_min_ = 2.3°
Absorption correction: multi-scan (*SADABS*; Sheldrick, 1996)	*h* = −23→21
*T*_min_ = 0.756, *T*_max_ = 0.862	*k* = −5→5
8859 measured reflections	*l* = −18→18

### Refinement

**Table:**

Refinement on *F*^2^	Primary atom site location: structure-invariant direct methods
Least-squares matrix: full	Secondary atom site location: difference Fourier map
*R*[*F*^2^ > 2σ(*F*^2^)] = 0.050	Hydrogen site location: inferred from neighbouring sites
*wR*(*F*^2^) = 0.140	H-atom parameters constrained
*S* = 1.05	*w* = 1/[σ^2^(*F*_o_^2^) + (0.073*P*)^2^ + 0.7018*P*] where *P* = (*F*_o_^2^ + 2*F*_c_^2^)/3
2324 reflections	(Δ/σ)_max_ = 0.001
137 parameters	Δρ_max_ = 0.72 e Å^−^^3^
0 restraints	Δρ_min_ = −0.46 e Å^−^^3^

### Special details

**Table:**

Geometry. All e.s.d.'s (except the e.s.d. in the dihedral angle between two l.s. planes) are estimated using the full covariance matrix. The cell e.s.d.'s are taken into account individually in the estimation of e.s.d.'s in distances, angles and torsion angles; correlations between e.s.d.'s in cell parameters are only used when they are defined by crystal symmetry. An approximate (isotropic) treatment of cell e.s.d.'s is used for estimating e.s.d.'s involving l.s. planes.
Refinement. Refinement of *F*^2^ against ALL reflections. The weighted *R*-factor *wR* and goodness of fit *S* are based on *F*^2^, conventional *R*-factors *R* are based on *F*, with *F* set to zero for negative *F*^2^. The threshold expression of *F*^2^ > σ(*F*^2^) is used only for calculating *R*-factors(gt) *etc*. and is not relevant to the choice of reflections for refinement. *R*-factors based on *F*^2^ are statistically about twice as large as those based on *F*, and *R*- factors based on ALL data will be even larger.

### Fractional atomic coordinates and isotropic or equivalent isotropic displacement parameters (Å^2^)

**Table:**

	*x*	*y*	*z*	*U*_iso_*/*U*_eq_	
Cl1	0.66489 (3)	0.91685 (16)	0.86511 (4)	0.0207 (2)	
N1	0.57606 (11)	0.7565 (5)	0.56683 (14)	0.0198 (4)	
N2	0.77325 (10)	1.1953 (5)	0.79768 (12)	0.0146 (4)	
C1	0.50497 (13)	0.5852 (7)	0.54853 (16)	0.0193 (5)	
H1A	0.4649	0.7516	0.5505	0.023*	
H1B	0.5022	0.4137	0.5988	0.023*	
C2	0.60887 (13)	0.7696 (6)	0.65240 (16)	0.0182 (5)	
H2	0.5878	0.6618	0.7016	0.022*	
C3	0.67969 (12)	0.9510 (6)	0.67619 (16)	0.0163 (5)	
C4	0.71175 (12)	1.0337 (6)	0.77157 (15)	0.0156 (5)	
C5	0.71871 (12)	1.0545 (6)	0.60569 (15)	0.0159 (5)	
H5	0.6999	1.0076	0.5407	0.019*	
C6	0.78589 (12)	1.2285 (6)	0.62878 (14)	0.0144 (5)	
C7	0.82776 (13)	1.3369 (6)	0.55873 (15)	0.0161 (5)	
H7	0.8108	1.2934	0.4930	0.019*	
C8	0.89247 (13)	1.5042 (6)	0.58613 (15)	0.0176 (5)	
H8	0.9209	1.5741	0.5391	0.021*	
C9	0.91795 (12)	1.5753 (6)	0.68373 (15)	0.0163 (5)	
H9	0.9630	1.6950	0.7008	0.020*	
C10	0.87912 (12)	1.4754 (6)	0.75412 (15)	0.0142 (5)	
C11	0.81194 (12)	1.2958 (6)	0.72724 (15)	0.0141 (5)	
C12	0.90516 (13)	1.5567 (6)	0.85775 (15)	0.0181 (5)	
H12A	0.9508	1.6891	0.8637	0.027*	
H12B	0.9144	1.3457	0.8941	0.027*	
H12C	0.8672	1.6880	0.8829	0.027*	

### Atomic displacement parameters (Å^2^)

**Table:**

	*U*^11^	*U*^22^	*U*^33^	*U*^12^	*U*^13^	*U*^23^
Cl1	0.0217 (3)	0.0290 (4)	0.0120 (3)	−0.0042 (2)	0.0040 (2)	0.0005 (2)
N1	0.0181 (10)	0.0211 (11)	0.0184 (10)	−0.0028 (8)	−0.0021 (7)	0.0020 (8)
N2	0.0164 (10)	0.0186 (10)	0.0089 (8)	0.0010 (8)	0.0022 (7)	0.0005 (7)
C1	0.0185 (12)	0.0208 (12)	0.0176 (12)	−0.0030 (10)	−0.0003 (9)	−0.0001 (9)
C2	0.0184 (11)	0.0191 (12)	0.0168 (11)	−0.0001 (9)	0.0019 (8)	−0.0006 (9)
C3	0.0178 (11)	0.0160 (12)	0.0144 (10)	0.0022 (9)	−0.0001 (8)	−0.0012 (9)
C4	0.0187 (12)	0.0171 (12)	0.0114 (10)	0.0016 (9)	0.0031 (8)	0.0019 (8)
C5	0.0201 (12)	0.0172 (11)	0.0095 (9)	0.0020 (9)	−0.0009 (8)	−0.0008 (8)
C6	0.0182 (11)	0.0150 (11)	0.0093 (10)	0.0039 (9)	0.0005 (8)	−0.0002 (8)
C7	0.0200 (12)	0.0209 (12)	0.0071 (9)	0.0046 (9)	0.0009 (8)	0.0011 (8)
C8	0.0203 (12)	0.0221 (12)	0.0109 (10)	0.0038 (9)	0.0041 (8)	0.0018 (8)
C9	0.0172 (11)	0.0171 (12)	0.0141 (10)	0.0004 (9)	0.0006 (8)	0.0000 (9)
C10	0.0190 (11)	0.0145 (11)	0.0086 (9)	0.0031 (9)	0.0009 (8)	−0.0006 (8)
C11	0.0159 (11)	0.0163 (11)	0.0097 (10)	0.0029 (9)	0.0012 (8)	0.0007 (8)
C12	0.0194 (11)	0.0252 (13)	0.0088 (10)	−0.0030 (10)	−0.0005 (8)	−0.0018 (9)

### Geometric parameters (Å, °)

**Table:**

Cl1—C4	1.752 (2)	C6—C7	1.414 (3)
N1—C2	1.267 (3)	C6—C11	1.427 (3)
N1—C1	1.457 (3)	C7—C8	1.362 (3)
N2—C4	1.299 (3)	C7—H7	0.9500
N2—C11	1.372 (3)	C8—C9	1.417 (3)
C1—C1^i^	1.517 (4)	C8—H8	0.9500
C1—H1A	0.9900	C9—C10	1.372 (3)
C1—H1B	0.9900	C9—H9	0.9500
C2—C3	1.477 (3)	C10—C11	1.422 (3)
C2—H2	0.9500	C10—C12	1.506 (3)
C3—C5	1.380 (3)	C12—H12A	0.9800
C3—C4	1.425 (3)	C12—H12B	0.9800
C5—C6	1.405 (3)	C12—H12C	0.9800
C5—H5	0.9500		
			
C2—N1—C1	117.8 (2)	C7—C6—C11	119.6 (2)
C4—N2—C11	117.47 (18)	C8—C7—C6	119.5 (2)
N1—C1—C1^i^	110.1 (2)	C8—C7—H7	120.2
N1—C1—H1A	109.6	C6—C7—H7	120.2
C1^i^—C1—H1A	109.6	C7—C8—C9	120.9 (2)
N1—C1—H1B	109.6	C7—C8—H8	119.5
C1^i^—C1—H1B	109.6	C9—C8—H8	119.5
H1A—C1—H1B	108.1	C10—C9—C8	121.6 (2)
N1—C2—C3	120.5 (2)	C10—C9—H9	119.2
N1—C2—H2	119.8	C8—C9—H9	119.2
C3—C2—H2	119.8	C9—C10—C11	118.47 (19)
C5—C3—C4	115.8 (2)	C9—C10—C12	121.8 (2)
C5—C3—C2	121.2 (2)	C11—C10—C12	119.77 (19)
C4—C3—C2	123.1 (2)	N2—C11—C10	118.43 (19)
N2—C4—C3	126.4 (2)	N2—C11—C6	121.7 (2)
N2—C4—Cl1	114.97 (16)	C10—C11—C6	119.9 (2)
C3—C4—Cl1	118.60 (18)	C10—C12—H12A	109.5
C3—C5—C6	120.9 (2)	C10—C12—H12B	109.5
C3—C5—H5	119.6	H12A—C12—H12B	109.5
C6—C5—H5	119.6	C10—C12—H12C	109.5
C5—C6—C7	122.61 (19)	H12A—C12—H12C	109.5
C5—C6—C11	117.8 (2)	H12B—C12—H12C	109.5
			
C2—N1—C1—C1^i^	−147.0 (3)	C11—C6—C7—C8	0.1 (3)
C1—N1—C2—C3	−177.6 (2)	C6—C7—C8—C9	−0.9 (3)
N1—C2—C3—C5	−12.4 (4)	C7—C8—C9—C10	0.8 (4)
N1—C2—C3—C4	167.0 (2)	C8—C9—C10—C11	0.1 (3)
C11—N2—C4—C3	−0.4 (4)	C8—C9—C10—C12	−178.7 (2)
C11—N2—C4—Cl1	−179.41 (16)	C4—N2—C11—C10	179.5 (2)
C5—C3—C4—N2	0.1 (4)	C4—N2—C11—C6	0.4 (3)
C2—C3—C4—N2	−179.3 (2)	C9—C10—C11—N2	179.8 (2)
C5—C3—C4—Cl1	179.03 (17)	C12—C10—C11—N2	−1.3 (3)
C2—C3—C4—Cl1	−0.3 (3)	C9—C10—C11—C6	−1.0 (3)
C4—C3—C5—C6	0.3 (3)	C12—C10—C11—C6	177.9 (2)
C2—C3—C5—C6	179.7 (2)	C5—C6—C11—N2	0.1 (3)
C3—C5—C6—C7	179.6 (2)	C7—C6—C11—N2	−180.0 (2)
C3—C5—C6—C11	−0.4 (3)	C5—C6—C11—C10	−179.1 (2)
C5—C6—C7—C8	−180.0 (2)	C7—C6—C11—C10	0.9 (3)

Symmetry codes: (i) −*x*+1, −*y*+1, −*z*+1.

### Hydrogen-bond geometry (Å, °)

**Table:**

*D*—H···*A*	*D*—H	H···*A*	*D*···*A*	*D*—H···*A*
C1—H1a···N1^ii^	0.99	2.59	3.299 (3)	128

Symmetry codes: (ii) −*x*+1, −*y*+2, −*z*+1.
